# Hyaluronic acid and phospholipid interactions useful for repaired articular cartilage surfaces—a mini review toward tribological surgical adjuvants

**DOI:** 10.1007/s00396-017-4014-z

**Published:** 2017-02-13

**Authors:** Stanislaw Jung, Aneta Petelska, Piotr Beldowski, Wayne K. Augé, Tahlia Casey, Dominik Walczak, Krzysztof Lemke, Adam Gadomski

**Affiliations:** 1 Institute of Mechanical Engineering, University of Science and Technology, Bydgoszcz, PL Poland; 2 Institute of Chemistry, University in Bialystok, Bialystok, PL Poland; 3 Institute of Mathematics and Physics, University of Science and Technology, Bydgoszcz, PL Poland; 4 Department of Research and Development, NuOrtho Surgical, Inc., Boston, MA USA; 5 Department of Biology, Delaware State University, Dover, USA; 6 Biovico sp z.o.o, Gdynia, PL Poland

**Keywords:** Hydrogels, Micelles, Amphiphiles, Biomedical applications, Colloids, Nanocomposites

## Abstract

This mini review is focused on the emerging nexus between the medical device and pharmaceutical industries toward the treatment of damaged articular cartilage. The physical rationale of hyaluronic acid and phospholipid preparations as tribological surgical adjuvants for repaired articular cartilage surfaces is explored, with directions for possible new research which have arisen due to the therapeutic advance of the physiochemical scalpel. Because synovial joint lubrication regimes become dysfunctional at articular cartilage lesion sites as a result of the regional absence of the surface active phospholipid layer and its inability to reform without surgical repair, hyaluronic acid and phospholipid intra-articular injections have yielded inconsistent efficacy outcomes and only short-term therapeutic benefits mostly due to non-tribological effects. Parameters for hydrophobic-polar type interactions as applied to the lubricating properties of normal and osteoarthritic synovial fluid useful for repaired articular cartilage surfaces are discussed.

## Introduction

Articular cartilage has been traditionally viewed as a tissue type with poor healing potential, a reputation attributable to Hunter [1] and repeated by such notable authors as Paget [2] and Mankin [3]. With the advent of arthroscopy, Jackson [4] introduced the modern age of articular cartilage surgery which ultimately led to repair opportunities he had thought would be possible [5]. In the last two decades, the nature of articular cartilage as an unrepairable tissue has been questioned by Augé [6], who has repaired partial-thickness articular cartilage lesions with a physiochemical scalpel to the endpoint of phenotypically differentiated and physiologically responsive tissue that is a suitable substrate for surface active phospholipid layer (SAPL) reconstitution [7, 8]. This advance ushered in the era of early intervention to reduce disease burden, producing an emerging nexus between the medical device and pharmaceutical industries.

Four primary observations led to the development of the physiochemical scalpel [9]. First, synovial joints have a limited clearance potential of damaged tissue at their articulating surfaces, a deficit which in many other tissue types translates to a clinically observed poor healing capacity. Second, synovial fluid pH changes and adjacent superficial zone healing responses occur commensurate with articular cartilage damage, exhibiting the common behaviors of secondary intention wound healing shared by many tissue types. Third, damaged articular cartilage lacks an oligolamellar SAPL due to increased surface roughness similar to the damaged surfaces of other tissue types requiring boundary lubrication. And fourth, various arthroscopic replacement media alter the character of damaged articular cartilage differently but have no substantive effect on normal surfaces with an intact SAPL due to an exclusion zone which forms at hydrophilic biosurfaces. Recognizing that synovial fluid also functions as both an irrigant and a wound healing exudate, and that damaged tissue could be trait-targeted based upon SAPL absence, the physiochemical scalpel was created using alternating current redox magnetohydrodynamic technology, traditionally utilized to power submarines, semiconductors, and nano- and micro-channel pumping devices, in a device configuration suitable for the operating theater.

Achieving precision resection with a physiochemical scalpel has shed additional light on the interfacial properties of articular cartilage, including its clearance deficiency, the role of its surface-confined nano-assembly layer, the self-targeting protonation effects arthroscopic lavage exerts on damaged regions, and the compositional changes of synovial fluid commensurate with disease, all reflecting species-conserved wound healing mechanisms to which the physiochemical scalpel is a biomimic [6, 9]. Precision resection enables exploration into the therapeutic potential of preserved tissue, creating an opportunity to recruit tissue previously over-resected or iatrogenically damaged, including access to innate genomic control mechanisms responsible for tissue assembly and healing, reestablishing suitable substrates upon which articular cartilage surface properties can be reconstituted toward a better bearing surface, and therein creating more normal chemomechanotransductive environments for subadjacent tissue during physiologic loading to avoid pathologic phenotypic shifts.

The ability to surgically reestablish surface properties emphasizes the need to explore the use of tribological surgical adjuvants, like hyaluronic acid (HA) and phospholipid (PL) preparations [10], in articular cartilage restoration and synovial fluid normalization beyond palliative care [9]. This mini review focuses on HA hydrophobic-polar type interactions with PL as applied to the lubricating properties of normal and osteoarthritic synovial fluid useful for repaired articular cartilage surfaces.

### Synovial fluid

Current treatment of damaged articular cartilage is based upon the appreciation that synovial joints are organ systems. In this view, the synovial fluid uniquely combines the medical device and pharmaceutical industry approaches because the synovial fluid is removed during surgical treatment and replaced by solutions through which visualization is improved [9]. This replacement naturally portends introducing therapeutic agents to augment or influence the reconstitution of synovial fluid during surgical convalescence. Table 1 depicts the synovial fluid profile in different clinical conditions.

**Table 1:** Description of normal and osteoarthritic synovial fluid composition

Synovial fluid examination	Normal	Early osteoarthritis	Late osteoarthritis	Rheumatoid arthritis
Lubricin (μg/mL)	364	244	152	139
Phospholipids (nmol/mL)	314.2	643.8	758.8	877.7
Hyaluronic acid (mg/mL)	2.2	1.7	1.9	1.0
pH	7.3	7.8	8.1	6.8

The rheumatoid arthritis parameters have been presented for the comparative purpose as a non-monotonic trend. Data extracted from [11]

While Jackson [4] popularized arthroscopic wash-out of synovial fluid associated with damaged articular cartilage as a means for symptom improvement, Augé [6] used that wash-out media to create a physiochemical scalpel, while envisioning surgical adjuvants to assist with reestablishment of more normal synovial fluid profiles and lesion surfaces during surgical convalescence.

### Medical device approach to articular cartilage treatment

The lack of surgical options available to treat articular cartilage damage before full-thickness lesions developed remained a historical problem. The simple and intuitive desire to remove damaged articular cartilage without iatrogenic collateral damage so that a healthy wound bed could be produced was impossible because all tissue resection devices had resulted in volumetric and/or functional over-resection.^1^ Because of this limitation, many concluded that either the removal of damaged articular cartilage should be relegated to only the most egregious clinical occurrences or patients with articular cartilage damage should be relegated to receive palliative treatment. Both viewpoints mutually reinforced the use of imprecise resection devices to achieve palliation; and considering cost containment pressures, low-cost mechanical devices remain default favorites.

**Fig. 1:**
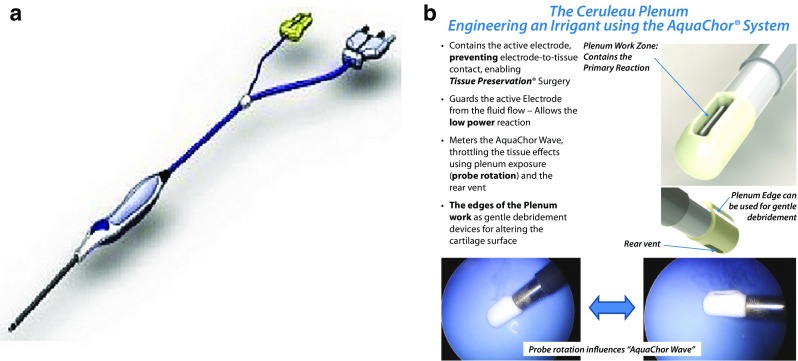
**a** Physiochemical scalpel. Ceruleau® (“blue water”) utilizes monopolar alternating current output to power a bipolar electrical device configuration to deploy higher voltage potentials and decreased current densities. This energy profile is optimal for fluid propulsion, producing a protonating guest exudate, generating increased extracellular matrix chondrocyte enrichment ratios, and inducing transcriptional upregulation of differentiated tissue assembly biomarkers. **b** Physiochemical scalpel mechanism of action. A protonating guest exudate, the AquaChor® System, is produced within a plenum primary reaction zone by powering the electrodes in a saline solution. The AquaChor Wave is delivered to the treatment site by surgical manipulation enabling tissue preservation surgery. Reproduced with permission from NuOrtho Surgical, Inc.; Boston, Massachusetts, USA

Figure 1 depicts the physiochemical scalpel as developed for the treatment of partial-thickness articular cartilage lesions. It functions as a biomimic of the respiratory burst myeloperoxidase system of polymorphonuclear neutrophil granulocytes at azurophilic degranulation without enzyme system deployment, delivering the primary reaction product of protonation potentials which are intimately involved in the acute phases of secondary intention wound healing. These protonation potentials are delivered to the tissue via an alternating current redox magnetohydrodynamic propulsive force through the structural diffusion of protons in aqueous media synovial fluid replacement environments that occur at pico- to femto-second rates (versus heat propagation which is comparatively very slow) as a “guest exudate” (Engineered Irrigant®) disaggregating damaged tissue not protected by an oligolamellar SAPL. The disaggregated damaged tissue becomes preconditioned for removal by gentle shear debridement in a manner similar to that which occurs during normal joint function that address smaller surface asperities. The protonation effect is accompanied by attendant temperature changes within the replacement media on the order of 2–5 °C that are similar to secondary wound healing exudates [7].

Although the responsive tissue subadjacent to articular cartilage lesions is a therapeutic target for the physiochemical scalpel, it remains collateral damage for all other tissue resection technologies. This collateral damage has been continuously problematic as surface phospholipid constructs (~450 nm) and the superficial zone (~200 μm) at or around articular cartilage lesions, and where many healing mechanisms and phenotypes reside, are always effectively eliminated, converting these areas into transitional or deep zone lesions. Precision resection with the physiochemical scalpel is a tissue rescue wound healing procedure that creates a differentiated and responsive wound bed demonstrating decreased surface roughness amenable to SAPL self-reconstitution, thereby inducing more appropriate chemomechanotransductive environments to avoid subadjacent pathologic phenotype shifts. Utilizing the technique of charge injection and migration, the electromagnetic forces accompanying higher voltage potentials induce non-transcription factor dependent and non-temperature dependent transcription initiation at promoter domains associated with tissue assembly gene clusters within non-over-resected, residually responsive wound sites of preserved tissue subadjacent to articular cartilage lesions.

### Pharmaceutical approach to articular cartilage treatment

Because the SAPL is absent at damaged articular cartilage surfaces, little attention has been afforded to the actual lubrication regimes in designing therapeutic agents because only until recently has surgical treatment been able to create a suitable substrate upon which to reconstruct effective lubrication assemblies and the resultant chemomechanotransductive improvements at and around lesion sites. Yet notwithstanding the foregoing, due to widely favorable biocompatibility profiles and the physiologic importance in synovial joint organ systems, HA and PL have been administered via intra-articular injection to provided palliative relief for articular cartilage damage.

**Fig. 2:**
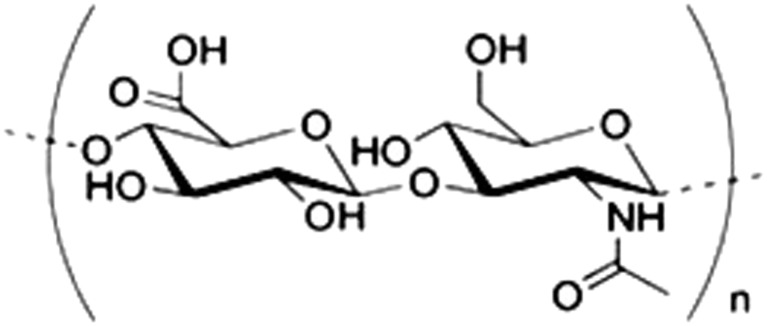
Hyaluronic acid monomer. HA is composed of repeating units of (1 → 4)*β* linked d-glucuronic acid and (1 → 3)*α* linked *N*-acetyl-d-glucosamine and can consist of up to 25,000 monomers

**Fig. 3:**
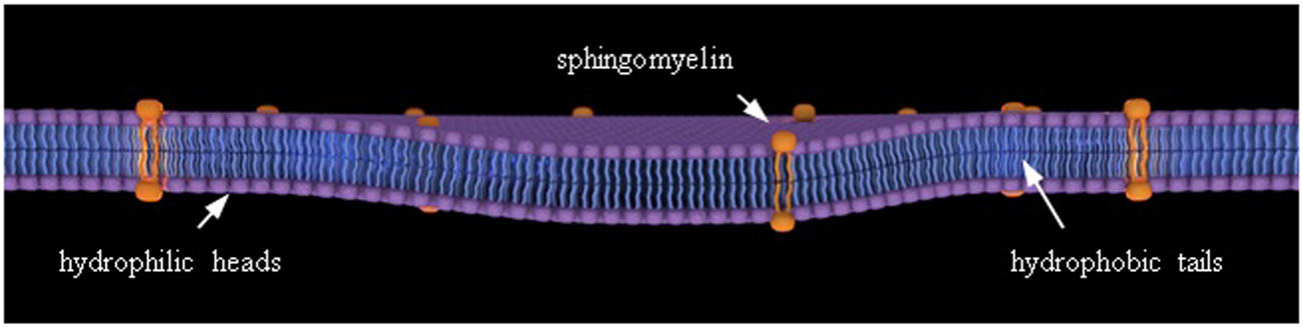
Phospholipid bilayer. The bilayer is composed of hydrophilic heads and hydrophobic tails such as that of phosphatidylcholine and phosphatidylethanolamine observed in the SAPL of articular cartilage. The orange amphiphiles represent SAPL sphingomyelin rafts considered to be involved in articular cartilage prototropic communication between the synovial fluid, SAPL, extracellular/pericellular matrix, pluripotential stem cells, and chondrocytes. Figure created by authors from data in [12]

HA is a polysaccharide (Fig. 2) present in the skin, synovial fluid, vitreous body of the eye, and components of body fluids and tissues and contributes to the optimal functioning of many processes and biological systems. HA adjusts synovial fluid viscosity and articulating surface lubrication, improves articular cartilage nutrition, and mediates cell growth regulation including proliferation, differentiation, and migration [11]. PLs comprise a diverse class of lipid molecules with amphiphilic properties which can form structures, including bilayers as shown in Fig. 3, which demonstrate surfactant properties when absorbed onto bearing surfaces [12]. In synovial joints, PLs are absorbed onto normal articular cartilage surfaces forming an oligolamellar SAPL which provides lubrication, force mitigation, and wound healing behaviors [7–9, 12]. For example, external perturbation forces typically experienced by articular cartilage facilitate intra- and interlamellar proton transport within the SAPL-producing asymmetric curvature forces that resist the inciting external perturbation [12].

Palliative treatment for early osteoarthritic degeneration has included intra-articular HA injections to aid in pain management and improvement of joint function over a period of months [13]. HA injections appear to provide clinical benefit by influencing chondrocyte and leukocyte function, proteoglycan metabolism, anti-inflammatory and analgesic mechanisms, and mechanical surroundings [14]. Yet, efficacy remains questionable for many reasons, including patient cohort non-homogeneity, different HA preparations (i.e., concentration, average molecular weight, rheological properties, volume), and commercial funding [15, 16]. The efficacy of HA treatment for lubrication is limited as exemplified by low rates of improved function and mobility as articular cartilage damage increases [17], and, when used in late stage osteoarthritis, efficacy is nearly indistinguishable from placebo [18]. Intra-articular injections of PL have been considered as a surfactant treatment modality with similar evidence of short-term symptomatic improvement [19–21]. The short-term results are largely independent of actual tribological properties because damaged articular cartilage surfaces are not a suitable substrate for adhesion and reconstitution of lubrication regimes without surgical repair first.

### Considerations for hyaluronic acid and phospholipid as tribological surgical adjuvants

#### Hyaluronic acid polydispersity

The sum of Gauss distribution can be used to describe the polydispersity of HA molecular mass as observed in varied synovial fluid conditions reflecting different viscoelastic properties evident in disease states [22]. The distribution function takes on the following form:

1 $$ f\left( x|\mu, {\sigma}^2\right)=\sum_{i=1}^N\frac{1}{\sqrt{2{\sigma_i}^2\pi}}{e}^{\frac{{\left( x-{\mu}_1\right)}^2}{2{\sigma_1}^2}} $$

where μ and σ^2^ are the mean and variance, respectively.

**Fig. 4:**
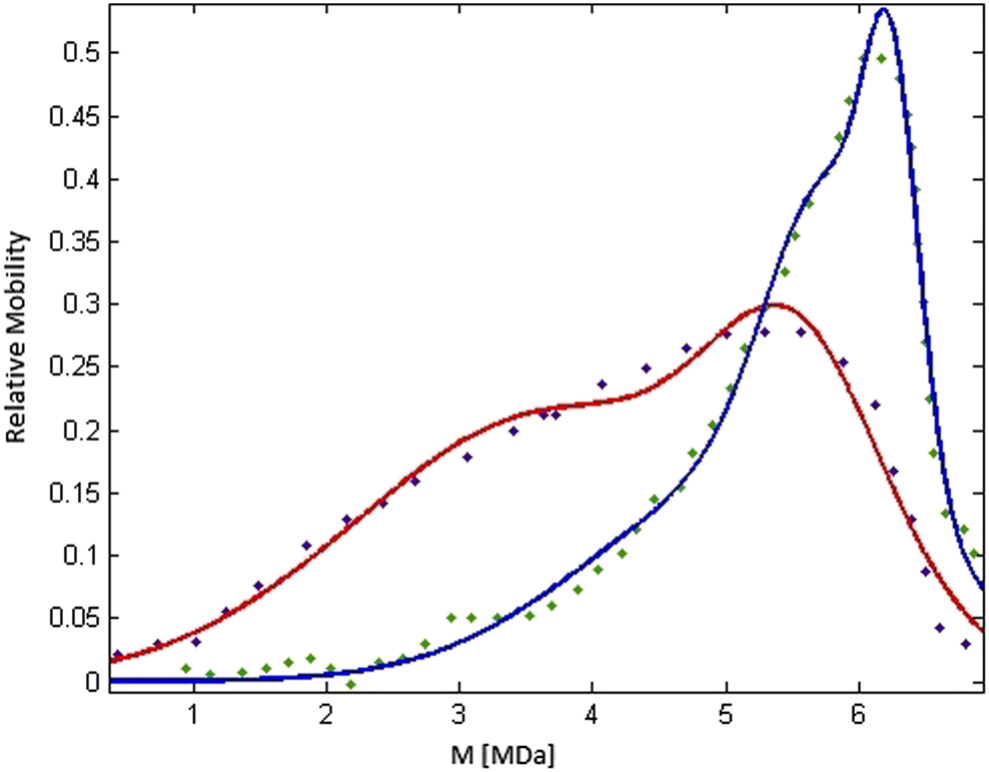
HA polydispersity in normal (*blue*) and osteoarthritic (*red*) synovial fluid as demonstrated by relative mobility in agarose gel electrophoresis [24]. Using readily available experimental data concerning HA polydispersity [22], Matlab CFTool (MathWorks; Natick, Massachusetts) and Digitizer software (Digizelt; Braunschweig, Germany) were used to match data of HA polydispersity with synovial fluid characteristics. The Levenberg-Marquardt algorithm was employed for non-linear least square curve fitting. The relative mobility was obtained from densitometry measurements, and molecular weight distribution is based on electrophoretic analysis of hyaluronan standards with known average molecular weight. The figure created from data in [22, 25]

As depicted in Table 2 and Fig. 4, HA polydispersity is altered in the diseased state of osteoarthritis. While normal synovial fluid contains predominantly long molecular HA chains, osteoarthritic synovial fluid contains shorter molecular HA chains reflective of the synovial organ system’s initial adaptation, and later dysfunction, in HA molecular population maintenance. Because the polydispersity profile in osteoarthritic synovial fluid is greatly variable based on disease stage, and most always contains a small but variable amount of high molecular mass fractions, statistical significance can be a confounding study parameter.

**Table 2:** Sum of Gauss fitting parameters for HA polydispersity as observed in normal and osteoarthritic synovial fluid conditions. These distribution parameters can be applied to describe polydispersity of HA molecular mass reflective of synovial fluid disease states with altered viscoelastic properties even though the polydispersity profile in osteoarthritis is highly variable

Synovial fluid condition	Medium [MDa]	Variance [Mda]	*R* ^2^
Normal	5.65	1.17	0.99
Osteoarthritis	4.24	1.74	0.97

Because large HA polydispersity is absent from healthy synovial fluid which contains only a very small fraction of molecules with less than 2 MDa molecular mass, therapeutic efforts have sought to normalize synovial fluid by injecting preparations with long chain molecules with larger molecular weight [23, 24]. While short-term symptomatic improvement has been demonstrated with intra-articular HA injections, the approach toward normalizing the HA polydispersity profile in osteoarthritic synovial fluid has not provided reliable symptomatic improvement. And as depicted in Table 1 above, synovial fluid pH is different in different clinical disease presentations, so that the pH effect on HA stability has been considered a contributor of the low efficacy of intra-articular HA injections. However, Maleki et al. [25] demonstrated that HA chain degradation occurs at pH < 4 and pH > 11, while virtually, no HA chain disruption occurs in the pH range 4–11. This pH-induced chain disruption is attributable to the cleavage of glycosidic bonds which further impairs network formation and the resultant alterations in viscoelastic behavior of synovial fluid.

#### Hyaluronic acid radius of gyration

The radius of gyration (R_g_) describes an average radius of molecules and is defined as follows:

2 $$ {R}_g^2=\frac{1}{N}\sum_{i=1}^N{\left({r}_i-{r}_{mean}\right)}^2 $$

**Fig. 5:**
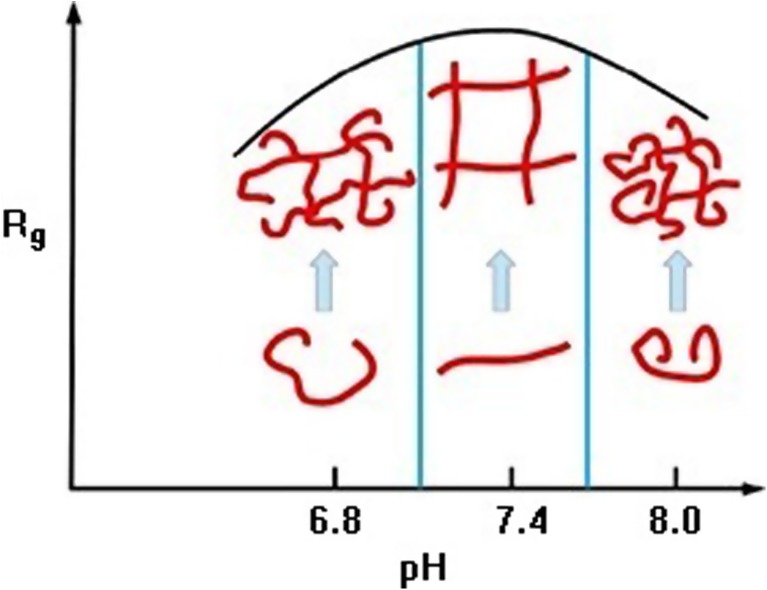
Qualitative depiction of radius of gyration of HA networks as a function of pH in normal (7.3), osteoarthritic (8.1), and rheumatic (6.8) synovial fluid. Figure created by authors from data in [19] using preparations with variations in HA chain length

HA network formation as a function of synovial fluid pH is depicted in Fig. 5 utilizing synovial fluid pH ranges observed clinically. As the radius of gyration attains its largest value, corresponding to a respective pH level, the resulting HA viscosity of the saline solution also takes on its largest value. These findings disclose an edge effect between viscosity and elasticity in which both effects are inseparable. The radius of gyration describes cross-linking efficiency, and hence, the higher its value reflects a better chance of association with other molecular chains toward network formation. Accordingly, at pH = 8.0, HA molecules are less able to create sustainable networks demonstrating sufficient tribological properties.

### Parameters for hyaluronic acid-phospholipid as tribological surgical adjuvants

In order to outline parameters for the preparation of tribological surgical adjuvants, a series of electrophoretic mobility measurement were performed with phosphatidylcholine liposomes containing HA to form a basis for investigation of ion/membrane association phenomena relevant to adhesion to repaired articular cartilage surfaces demonstrating healthy wound beds without abnormal surface asperities. The measurements were performed at several pH values using 0.155 M sodium chloride as the supporting electrolyte solution.

Liposomes were prepared by sonication. Phosphatidylcholine and hyaluronic acid were weighed, dissolved in chloroform (10 mg cm^−3^), and mixed in various molar ratios of PL/HA (3:1, 1:1, 1:3). HA of average molecular mass 1.6–2.0 MDa and a concentration of 22 mg/ml (BioVico®; Gdynia, Poland) and PL of 99% egg yolk L-α-Phosphatidylcholine (Sigma; St. Louis, MO) were used. The solvent was evaporated under a gentle stream of argon gas to obtain dry lipid film and thereafter, hydrated with sodium chloride solution (0.155 mol dm^−3^). Ultrasound disintegrator UD-20 (Techpan; Poland) with a “Sandwich” concentrator was used as an ultrasonic source, consisting of a power generator, ultrasonic vibration transducer, and a titanium tip sonotrode (diameter 12 mm and amplitude 16 μm). A maximum generator output power of 180 W and a vibration frequency of 22 kHz were deployed. Sonication was applied five times for 90 s. Since heat is liberated during the process, the suspension was cooled by using an ice bath of an ice and dry sodium chloride mixture. Liposome size was determined using a Zetasizer Nano ZS apparatus (Malvern Instruments, UK) and exhibited a distribution profile with one population (representing ~90% of all particles) of a 160 nm diameter and the other population (representing ~9% of all particles) of a 30 nm diameter.

Liposome mobility was determined by performing micro-electrophoretic assessments on samples and measuring the velocity of the particles using laser Doppler velocimetry with the Zetasizer Nano ZS apparatus. The measurements were performed as a function of pH. Formed liposomes were suspended in alkali metal chloride solution. To change the pH, the corresponding amount of acid or base was added. The reported values represent the average of at least six measurements performed at each pH value. All experiments were performed at least three times.

#### Surface charge density determination

Electrophoretic mobility values were converted to surface charge density using Eq. 3 [26]. From electrophoretic mobility measurements, the surface charge density was determined by

3 $$ \sigma =\frac{\eta \cdot u}{d}, $$

where *σ* is the surface charge density, *η* is the viscosity of solution, *u* is the electrophoretic mobility, and *d* is the diffuse layer thickness.

The diffuse layer thickness was determined from the formula [27]:

4 $$ d=\sqrt{\frac{{\varepsilon \varepsilon}_0 RT}{2{F}^2 I}}, $$

where *R* is the gas constant, *T* is the temperature, *F* is the Faraday’s constant, *I* is the ionic strength of the electrolyte, *ε* is the permeability, and *ε*_o_ is the absolute value of permeability of the electric medium.

**Fig. 6:**
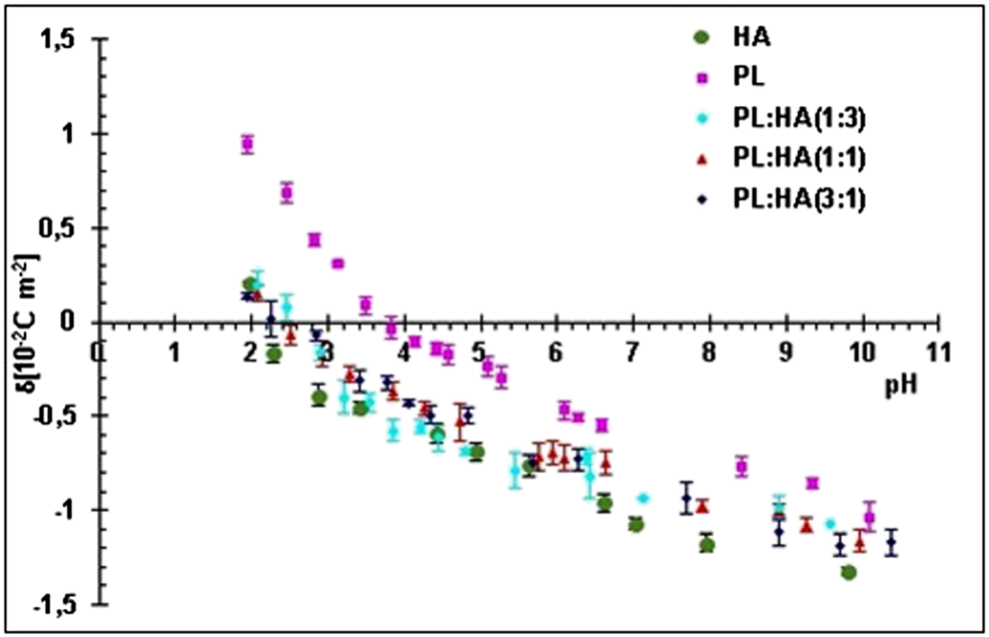
The pH dependence of the surface charge density of the liposomal membranes created by HA, PL, and HA/PL mixture in sodium chloride solution

Figure 6 shows that HA exhibits a positive charge only at the most acidic pH, and when the solution becomes more basic, the surface charge of HA remains negative. The isoelectric point compared to that of phosphatidylcholine liposomes shifts from pH 3.8 to 2.2. In the range of physiologic conditions of synovial fluid pH (6.8 to 8.1), the surface charge of HA vesicles increases by ~10% such that there is no significant change in the electrostatic properties of the HA net and therefore its ability to perform electrostatic cross-linking should not change due to its almost constant charge. Physiologic pH seems to be the set value for ideal hyaluronic acid interactions that best allow lipids to form micelles [28], which creates the optimal lubricating conditions within synovial fluid. HA/PL complexes exhibit slightly different behaviors depending on the composition, namely the ratio of PL to HA. The HA/PL complexes possess lower absolute values of surface charge than lone HA molecules.

#### HA/PL interactions

**Fig. 7:**
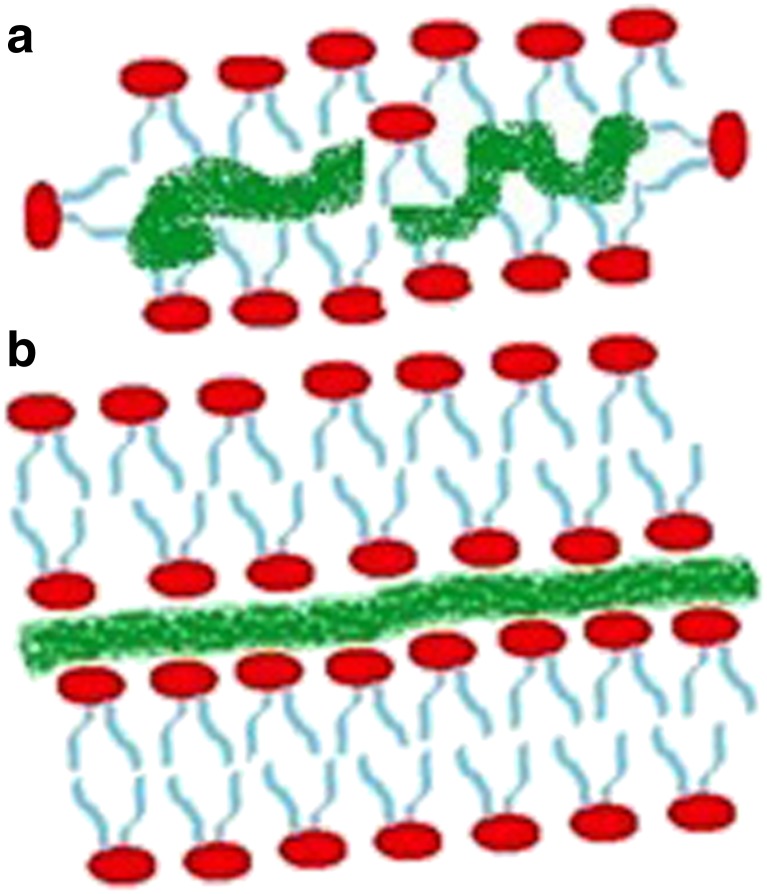
Graphical depiction of HA/PL complex conformation. The *wide green line* represents HA, whereas *red* and *blue parts* of PLs represent hydrophilic heads and hydrophobic tails, respectively. Two forms are depicted: **a** short HA absorbed at the interior of PL vesicle-cylindrical structures and **b** bilayer around HA-brush-like structures as in hydration-repulsion mechanisms. Figure created by authors from data in [28]

Long HA chains crosslink easier and create structural nets for lipidic bearings. A significant increase in the number of HA chains with molecular mass below 1 MDa leads to preferential absorption on PL vesicles rather than in forming cylindrical structures with phospholipid through crosslinking mechanisms [28] (Fig. 7).

Because long HA chains exhibit a higher probability of forming HA/PL cylindrical structures necessary for facilitated lubrication mechanisms, the finding that HA/PL complexes possess lower surface charge than lone HA molecules is consistent with the formation of brush-like structures.

## Discussion

### Emerging nexus between the medical device and pharmaceutical industries

**Fig. 8:**
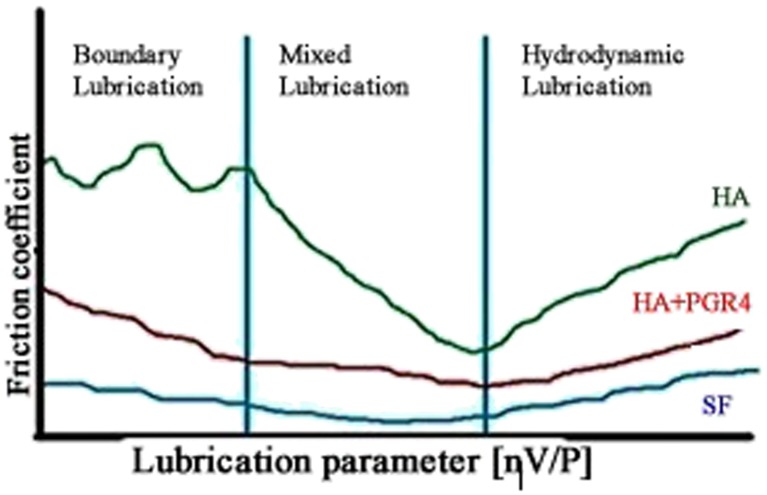
Lubrication regimes within synovial fluid presented in a Stribeck-type curve. The *red line* depicts HA plus PGR4; the *blue*, synovial fluid; and the *green*, HA. Adapted from [30]. Not shown in this depiction are phenomena such as elastohydrodynamic, boosted, and weeping lubrication commonly observed at opposing biological surfaces such as articular cartilage

Synovial joint lubrication has been divided into three general regimes as depicted in Fig. 8, namely boundary, mixed, and hydrodynamic. Although a gross over simplification of articular cartilage tribology, the depiction is useful in demonstrating demarcations between the solid surface asperities of boundary lubrication repaired by the physiochemical scalpel and the fluid film properties of hydrodynamic lubrication of interest to those considering formulation of true tribological surgical adjuvants. These lubrication regimes become dysfunctional at articular cartilage lesion sites due to the regional absence of the SAPL that serves to integrate tribology function of the different states of matter in synovial organ systems. Because partial thickness articular cartilage lesions are repaired during synovial fluid replacement with saline solutions, an interfacial milieu is produced which exhibits hydrodynamic fluid film starvation and favors the expression of boundary lubrication regimes, a situation ideal for treatment of abnormal surface asperities [9]. This media replacement has been shown to influence friction coefficients, but not proton channel transport time, and that divalent cations should be avoided to prevent interference with SAPL function [29]. Such interference is important to consider when pursuing post-repair SAPL reconstitution.

Synovial fluid replacement during surgical repair of articular cartilage lesions creates an ideal venue to reestablish synovial fluid composition and character after treatment as an adjuvant procedure. Because synovial fluid polydispersity and abnormal surface asperities are eliminated during surgical repair, solutions reestablishing pH normalized HA/PL complexes and delivered after surgical repair may enhance SAPL self-assembly at repair sites. These complexes can be delivered during surgical repair by magnetohydrodynamic propulsion within the synovial fluid replacement media such that appropriately configured molecular structures of tribological surgical adjuvants will self-assemble at the repair site. Proposed mechanisms for absorption and anchoring related to the HA/PL surface charge assessments presented in this mini review include hydrophobic or electrostatic association, ion exchange, and Langmuir–Blodgett deposition. While this mini review does not address absorption and desorption kinetics that may be enhanced over that which occurs as the native synovial fluid reforms during surgical convalescence, HA is an anionic polymer that can bind phospholipids such that pre-exposure of the repaired articular surface to various pH normalized HA/PL complexes within the protonating irrigant of the physiochemical scalpel can overcome the negative charge density of interstitial tissues that has served as a barrier to deposition during conventionally supplied HA and PL.

### HA and PL as tribological surgical adjuvants

According to Klein [31], facilitated lubrication arises due to hydration repulsion as well as a brush-like structural mechanism. The former mechanism occurs on phospholipid bilayers near normal articular cartilage surfaces as a boundary lubrication regime and also manifests in bulk synovial fluid as a result of hydrophobic-polar interactions in respective volumetric subspaces of articulating systems [32]. This hydration-repulsion mechanism may also be involved in the capacity to increase force field absorption as the hydrophilic heads repel one another, creating a type of cushioning effect supported by the corresponding screened electrostatic conditions [33]. Accordingly, reverse micelles can lower the friction coefficient by changing the friction mode in which a quasi-static friction effect can be replaced by its roll-over counterpart [30]. The latter mechanism occurs at boundary lubrication sites as a result of lubricin, O-linked glycoprotein, and inter-chain interactions between HA and phospholipids [34]. HA is a key synovial fluid component that provides full viscoelastic properties to the system. In bulk synovial fluid, HA forms networks which are involved in increasing load bearing capacity such that the cross-linking mechanisms strengthen the lubricating properties of synergistically interacting phospholipids. Figure 6 shows that in an osteoarthritic condition with pH shifted above pH 7.4, HA molecules display higher surface charge. This occurrence could be explained by smaller values of gyration radii in basic solution such that the surface area of HA micelles in basic pH is smaller than in neutral ones. These findings seem to correlate well with results of Maleki et al. who found the maximum value of HA molecular weight and radius of gyration in pH ~7.0 [25].

The interaction between phospholipids and the links between HA fibers result in cylindrical/micellar forms around these fibers to absorb the bulk force applied to articular cartilage and facilitate lubrication within the joint [32]. According to Pasquali-Ronchetti et al. [28], the facilitated lubrication in this regime is obtained by the brush-like lubrication mechanism whereby phospholipid heads attach to HA creating reverse cylindrical micelles. This hydration-repulsion mechanism may also be involved in the capacity to increase force field absorption as the hydrophilic heads repel one another, creating a type of cushioning effect supported by the corresponding screened electrostatic conditions [34] in addition to reactive membrane curvature forces discussed previously [12]. Consequently, reverse micelles can lower the friction coefficient by changing the friction mode in which a quasi-static friction effect can be replaced by its roll-over counterpart [35].

The findings of an edge effect between viscosity and elasticity in which both effects are inseparable create a key tool for qualitatively interpreting the overall involvement of synovial fluid viscoelasticity which is a pivotal effect on facilitated lubrication. The radius of gyration is a quantity of the same order as the value of HA molecule hydrodynamic radius with the measure of synovial fluid electrolytic properties represented as pH values. Additionally, as from the Einstein-Stokes formula, viscosity inversely depends on the hydrodynamic radius in the diffusion coefficient [35] so that the elastic sub-effect supports any mechanical loads applied on the articulating two-surface system with a synovial fluid viscoelastic interlayer. This sub-effect can be viewed as a nano-cushioning articular cartilage matrix effect. As a consequence, there exists only a well specified and narrow range of pH values which makes the lubrication really facilitated, cf. Fig. 5.

### In vivo therapeutic considerations

As juxtaposed surfaces interact, abnormal wear can increase lesion size causing increased concentration of lipids in synovial fluid by lesion site extrusion and ineffectual synthesis. In vivo, a lipid overcrowding effect may lower the facilitated lubrication effect. As the result of interplay between these two processes, different regimes appear in which the effective diffusion coefficient shows peculiar behaviors such as a decrease due to the presence of the adsorption process and a non-monotonous behavior resulting from particle interactions [36]. Further, biological membranes such as the SAPL carry electric charges of differing origin. Part of the charge fixed to the membrane originates from dissociation of the carboxyl groups of sialic acids. Other negative charges stem from several phospholipids. Other components also, with their cationic and anionic groups, contribute to the charge on the membrane. Protons are the charge-determining ions for all these groups, and the membrane charge density is therefore directly dependent on the pH. At essentially all physiological pH values, the net membrane charge is negative reaching values of a few to a few tens of C cm^−2^ [33]. The membrane surface charge density is an important parameter for characterizing equilibria in the membranes, and this parameter strongly depends on environmental conditions such as pH and membrane composition [37, 38].

Absorption of HA/PL complexes onto repaired articular cartilage surfaces to initiate reformation of the SAPL [39] can be expedited by surface charge modification to match the exposed articular cartilage interstitial matrix. Such modification can be obtained by inserting HA molecules in PL vesicles and modification of system pH. The PLs used in this mini review study have an isoelectric point at pH = 3.8, well beyond physiologic conditions, so that by modifying the PL contribution, the isoelectric point can be shifted toward more optimal values to improve absorption-desorption kinetics. Future work will consider modifications to create attractive absorption-desorption kinetics for articular cartilage lesions after repair and the role of hydrodynamic interactions [40].

## Conclusion

The successful treatment of lesions before the point-of-no-return is that which can positively alter disease burden, and the capabilities of the physiochemical scalpel have challenged articular cartilage stakeholders to seek additional non-palliative solutions for early intervention. This mini review is an initial assessment toward new tribological surgical adjuvant methods that are directed away from palliative care and toward improvement of articular cartilage lesion site chemomechanotransductive properties associated with better-quality lubrication. Such adjuvant methods provide an opportunity to influence synovial fluid performance by modifying viscoelasticity and absorption-desorption kinetics of HA/PL complexes. Because conventional injections of HA or PL have failed to act as a lubricant for unrepaired articular cartilage lesions due to unfavorable synovial fluid pH, distorted synovial fluid composition, and abnormal surface asperities that can switch the lubrication mode uncontrollably from boundary to mixed or even hydrodynamic, this dysfunction can cause severe wear conditions to occur, among which includes further loss of effective lubricating material. Additional focus on the pH influence on gyration radii, surface asperity height control, and absorption-desorption kinetics is a special future task worth addressing in the non-palliative treatment of articular cartilage lesions.

## Footnotes

1 Prior to the physiochemical scalpel, the most precise endoscopic tissue resection devices had been thermal and plasma ablation devices. Although more precise than mechanical shavers, these devices create at least 200–250 μm of collateral damage in addition to the tissue resected and are a palliative approach because lesion size is expanded.
